# Real-World Molecular Biomarker Testing Patterns and Results for Advanced Gastroesophageal Cancers in the United States

**DOI:** 10.3390/curroncol30020145

**Published:** 2023-02-03

**Authors:** Rutika Mehta, Astra M. Liepa, Shen Zheng, Anindya Chatterjee

**Affiliations:** 1 Moffitt Cancer Center, Tampa, FL 33612, USA; 2 Eli Lilly and Company, Indianapolis, IN 46285, USA; 3 TechData Service Company, King of Prussia, PA 19406, USA

**Keywords:** gastroesophageal cancers, biomarker testing patterns, treatment sequences

## Abstract

The decision to treat advanced gastroesophageal cancers (GECs) with targeted therapy and immunotherapy is based on key biomarker expression (human epidermal growth factor receptor 2 (HER2), programmed cell death-ligand 1 (PD-L1), microsatellite instability (MSI), and/or mismatch repair (MMR)). Real-world data on testing, results, and treatment patterns are limited. This retrospective observational study used a nationwide electronic health record-derived de-identified database of patients from the United States. The analysis included adult patients with advanced GECs who initiated systemic treatment between 2017 and 2020. Biomarker testing patterns, timing, assays, tissue collection site, results, and treatment sequences were assessed. Of 1142 eligible patients, adenocarcinoma was the most prevalent histology (83% of patients). Overall, 571 (50%) patients were tested for PD-L1, 582 (51%) were tested for MMR/MSI, and 857 (75%) were tested for HER2. Between 2017 and 2020, the PD-L1 testing rate increased from 39% to 58%, and the MMR/MSI testing rate increased from 41% to 58%; the median time from initial diagnosis to first test decreased for both biomarkers. Programmed cell death receptor-1 inhibitor use was observed among patients with positive PD-L1 or MMR-deficient/MSI-High results. These results supplement data reported in key clinical trials and may inform decision-making as treatment options for advanced GECs evolve.

## 1. Introduction

In 2020, gastric and esophageal cancers had among the highest incidence and mortality rates globally [1]. In 2022, in the United States, an estimated 26,380 new cases of gastric cancer and 20,640 new cases of esophageal cancer were expected to be reported [2,3]. Cancers of the esophagus, gastroesophageal junction (GEJ), and stomach (collectively called gastroesophageal cancers (GECs)) account for about 2.5% of all new cancer diagnoses but are the seventh leading cause of cancer-related mortality in the United States [4].

The relatively aggressive nature of GECs and lack of regular screening practices may contribute to their late detection and poor prognosis [5]. Consequently, curative interventions cannot be offered in most cases. Despite major advances in the field, median survival for metastatic disease is about 1 year [6].

The backbone of standard first-line (1L) treatment for advanced GECs is systemic chemotherapy [7,8]. The addition of targeted therapy and/or immunotherapy is informed by biomarker expression. Therefore, it is important to assess biomarker status at treatment initiation [9]. Some notable biomarkers in GECs are human epidermal growth factor receptor 2 (HER2), programmed cell death-ligand 1 (PD-L1), microsatellite instability (MSI), or mismatch repair (MMR) [10].

After the results of the Trastuzumab for Gastric Cancer (ToGA) trial in 2010, HER2 testing became standard practice for GECs [11]. In 2017, the United States Food and Drug Administration (FDA) granted accelerated approval of the programmed death receptor-1 (PD-1) inhibitor pembrolizumab for refractory PD-L1-positive gastric cancers [12], although the approval was withdrawn in 2021 [13]. A recent multi-trial analyses emphasized the importance of MSI and MMR as biomarkers in GECs [14]. In 2021, the FDA approved 1L chemoimmunotherapy combinations based on positive data from KEYNOTE-590 and CHECKMATE-649 [15,16,17,18]. While approvals were not based on stringent biomarker cut-offs, most experts use PD-L1 cut-offs when considering adding immunotherapy to chemotherapy in HER2-negative patients. Use of immunotherapy in GEC treatment is based on primary tumor location, line of therapy (LOT), histology, and in some cases, MMR/MSI status and PD-L1 expression as measured by combined positive score (CPS) [19].

Limited real-world data, especially in community practice, are available for biomarker testing patterns, particularly for more recently introduced biomarkers in advanced GECs. This study sought to describe real-world testing patterns in the United States for biomarkers (PD-L1, MMR/MSI, and HER2) and the use of next-generation sequencing (NGS) in advanced GECs. The study also described treatment sequences in the context of biomarker testing.

## 2. Materials and Methods

### 2.1. Study Design and Patients

This was a retrospective observational cohort study conducted using the nationwide Flatiron Health electronic health record (EHR)-derived de-identified database [20].

The Flatiron Health database is a longitudinal database comprising patient-level structured and unstructured data, curated via technology-enabled abstraction [20,21]. The Flatiron Health Advanced Gastric/Esophageal Cancer Cohort includes patients diagnosed with advanced GECs from approximately 280 cancer clinics (approximately 800 sites of care) in the United States. The database includes patient demographics, practice setting, diagnosis, staging, histology, HER2 biomarker testing and status, and systemic therapies. Biomarker results are abstracted from laboratory reports with no additional interpretation.

Patients in this cohort were diagnosed with recurrent or metastatic disease on or after 1 January 2011 and had ≥2 visits on different days documented in the EHR. Patients had advanced GEJ/esophageal or gastric cancer (International Classification of Diseases, Ninth Revision (ICD-9) 150.x or 151.x or ICD, Tenth Revision (ICD-10) C15.x or C16.x), with evidence and pathology consistent with esophageal, GEJ, or gastric cancer.

Additional data abstraction was conducted to supplement the existing database, including PD-L1 and MMR/MSI testing. If CPS was available, it was reported categorically using ranges. NGS testing for any panel (yes versus no) and as an assay method for HER2 and MSI were reported. This additional abstraction was conducted for a random sample of approximately 1500 patients who initiated 1L therapy between 1 January 2017 and 31 August 2020. These patients represented the primary study cohort for whom study-specific eligibility criteria were applied.

Patients aged ≥18 years with ≥3 months of follow-up from the start of 1L treatment (i.e., initiated by 1 June 2020) were included. Patients who received any treatment as part of a clinical trial and those with no evidence of first activity within 3 months of advanced diagnosis date were excluded.

The index date was defined as the date of the start of 1L therapy.

The study dataset was created under an institutional review board-approved protocol (WIRB-Copernicus Group institutional review board protocol #420180044) compliant with the United States patient confidentiality requirements, including the Health Insurance Portability and Accountability Act (HIPAA) of 1996 regulations.

### 2.2. Outcomes

Baseline demographic characteristics relative to the start of 1L therapy were summarized. Disease characteristics, biomarker testing (PD-L1, MMR/MSI, and HER2), and NGS testing were described. The number of biomarker tests per patient, assay types, testing combinations, and timing were described. Testing results (positive, negative, or unknown) were summarized. Treatment sequences for up to 3 LOTs by testing results were described, highlighting the use of PD-1 inhibitors and HER2-targeted agents.

Regimens and LOTs were derived [22]. Hormonal agents were excluded. As radiotherapy is not available in the database, regimens of weekly carboplatin and paclitaxel were assumed to be in conjunction with radiotherapy and were excluded as LOTs.

### 2.3. Statistical Analysis

All analyses were descriptive. Patients were classified based on evidence of biomarker testing (tested versus not tested) and by testing results. Patients were further grouped by primary tumor site and histology and by year when 1L therapy started.

For categorical variables, summary statistics were presented as frequencies and percentages. Continuous variables were presented as median (range, interquartile range (IQR)). No imputations were made for missing data. Missing data were excluded from analyses. Time on treatment was estimated using the Kaplan–Meier method. Treatment was censored at the end of the database if the last administration was within 21 days of the end of the database and the patient was alive.

Statistical analyses were performed using SAS Enterprise Grid, Version 7.15. Box plots and Sankey diagrams were generated using R, Version 4.1.0 [23].

## 3. Results

Additional data abstraction was conducted for 1594 patients with advanced GECs who were selected from the database based on the specified timeline. Of these patients, 1142 patients met study-specific eligibility (Figure S1).

### 3.1. Demographics and Baseline Characteristics

Most of the population (>60%) was ≥65 years of age and predominantly male (75%) and White (64%). Most patients were treated at community clinics (92%; Table S1).

Nearly 50% of patients had esophageal cancer, 30% had gastric cancer, and 22% had GEJ cancer. Approximately 83% of patients had adenocarcinomas. Nearly 88% of patients were initially diagnosed with advanced or metastatic GECs, and median follow-up was about 9 months (Table 1).

#### PD-L1, MMR/MSI, and HER2 Testing

Overall, 571 (50%) patients were tested for PD-L1, 582 (51%) were tested for MMR/MSI (Table S1), and 857 (75%) were tested for HER2 (Table S2). NGS was performed for 394 patients (35%; Table S3).

For all biomarkers, patients who were tested were younger than those who were not tested. The median (range) age was 67 (27–84) years and 70 (23–84) years for patients tested and not tested for PD-L1, respectively, and 66 (27–84) years and 70 (23–84) years for patients tested and not tested for MMR/MSI, respectively (Table S1). The median (range) age was 67 (23–84) years and 70 (38–84) years for patients tested and not tested for HER2, respectively (Table S2).

### 3.2. Overall Biomarker Testing Details

Assays were performed prior to 1L therapy in 499 (87%) patients tested for PD-L1 and in 514 (88%) patients tested for MMR/MSI.

Over 70% of PD-L1 tests were conducted using the commercial 22C3 assay. Approximately 46% of MMR/MSI testing was performed using immunohistochemistry and 43% using NGS. Approximately 75% of PD-L1 tests and 68% of MMR/MSI tests were conducted using tissue from the primary tumor site (Table S4).

The median (range) number of tests per patient was 1 (1–4) for PD-L1 testing and 1 (1–6) for MMR/MSI testing. Among patients with multiple tests, the first two tests were performed on the same day in 68% of cases for PD-L1 and in 70% of cases for MMR/MSI. The same assay type was used for PD-L1 testing for 87% of patients. Different test types were used for MMR/MSI tests for 75% of patients (Table S5). 

**Figure 1:**
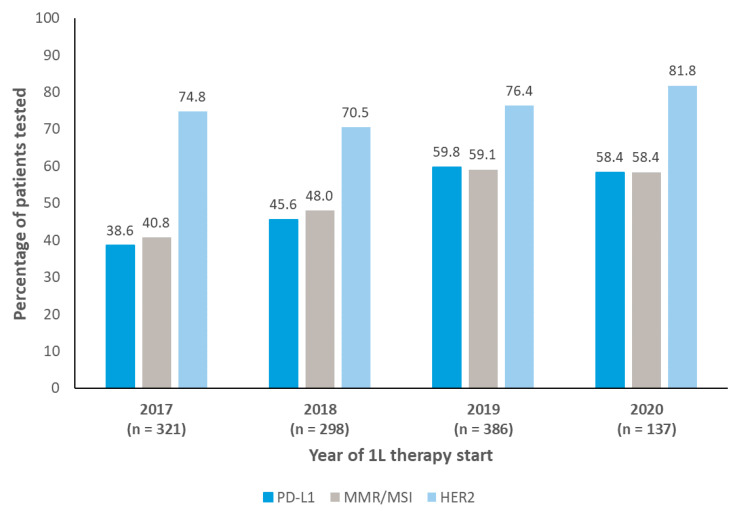
Biomarker testing by year of 1L therapy start. 1L, first-line. HER2, human epidermal growth factor receptor 2. MSI, microsatellite instability. MMR, mismatch repair. n, number of patients tested. PD-L1, programmed cell death-ligand 1.

PD-L1 and MMR/MSI testing rates increased from 2017 to 2020 (from 39% to 58%, and 41% to 58%, respectively; Figure 1).

**Figure 2:**
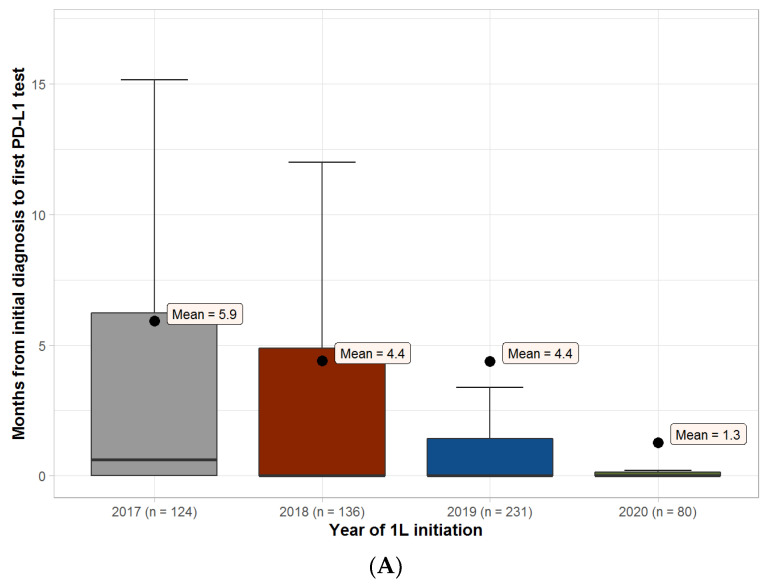

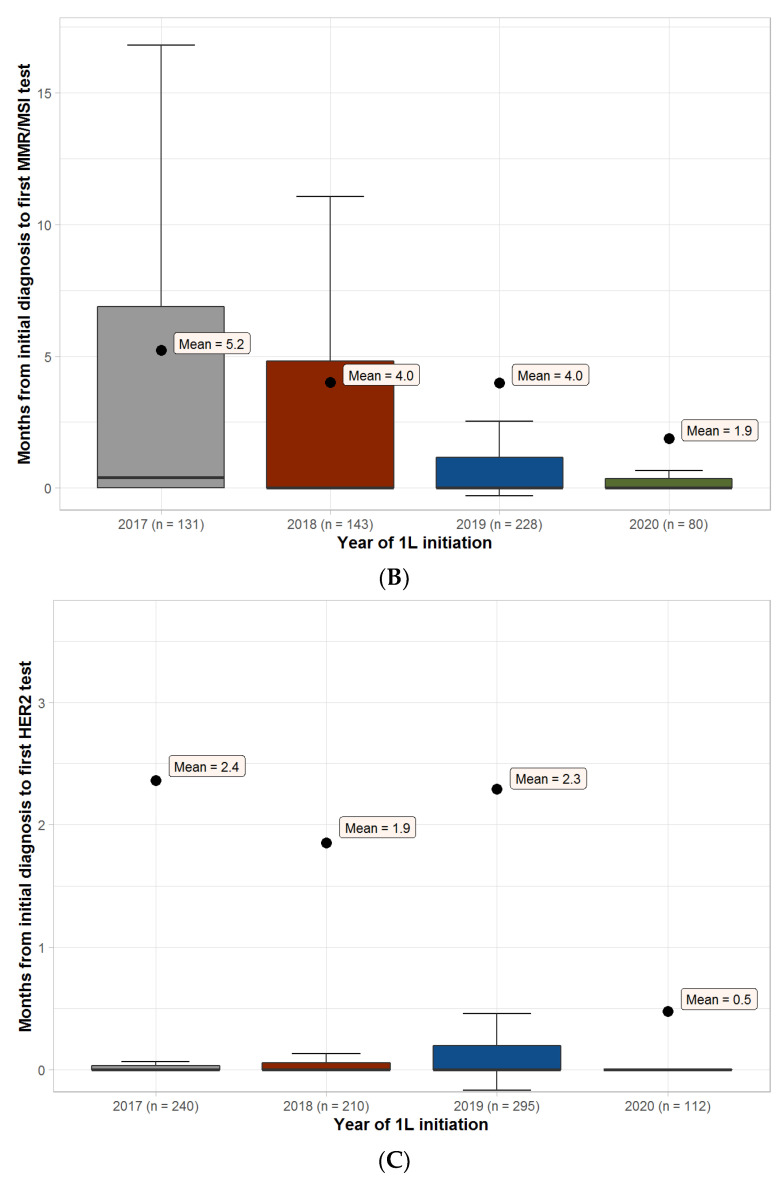
Months from initial diagnosis of gastroesophageal cancer to first biomarker test. Boxplots of time (months) from initial diagnosis of gastroesophageal cancer to first biomarker test. Boxes represent the interquartile range (IQR), and the thick horizontal line represents the median (this value is 0 for years 2018–2020 for all biomarkers). The upper whisker represents (Q3 + 1.5xIQR), and the lower whisker represents (Q1 − 1.5×IQR). The outliers are not shown on the graph. 1L, first-line. HER2, human epidermal growth factor receptor 2. MSI, microsatellite instability. MMR, mismatch repair. n, number of patients. PD-L1, programmed cell death-ligand 1. (**A**) PD-L1; (**B**) MMR/MSI; (**C**) HER2.

The median (IQR) time from initial GEC diagnosis to first biomarker test was 0.6 (0–6.3) months in 2017 and 0 (0–0.1) months in 2020 for PD-L1, 0.4 (0–7.1) months in 2017 and 0 (0–5.1) months in 2020 for MMR/MSI, and 0 (0–0) months in both 2017 and 2020 for HER2 (Figure 2).

Of the 571 patients tested for PD-L1, 117 (20%) tested positive and 357 (63%) had unknown results. Of the 582 patients tested for MMR/MSI, 30 (5%) tested MMR-deficient (dMMR)/MSI-High (MSI-H; Figure S2). dMMR/MSI-H results were only observed for adenocarcinoma histology.

Of the 571 patients tested for PD-L1, 406 (71%) had CPS reported. Of these, 78 (19%) patients had CPS < 1 and 200 (49%) had CPS ≥ 5. CPS varied by primary tumor site and histology (Table S6). Table S7 presents the clinical and disease-related characteristics by HER2 testing status.

#### Biomarker Testing Combinations 

**Figure 3:**
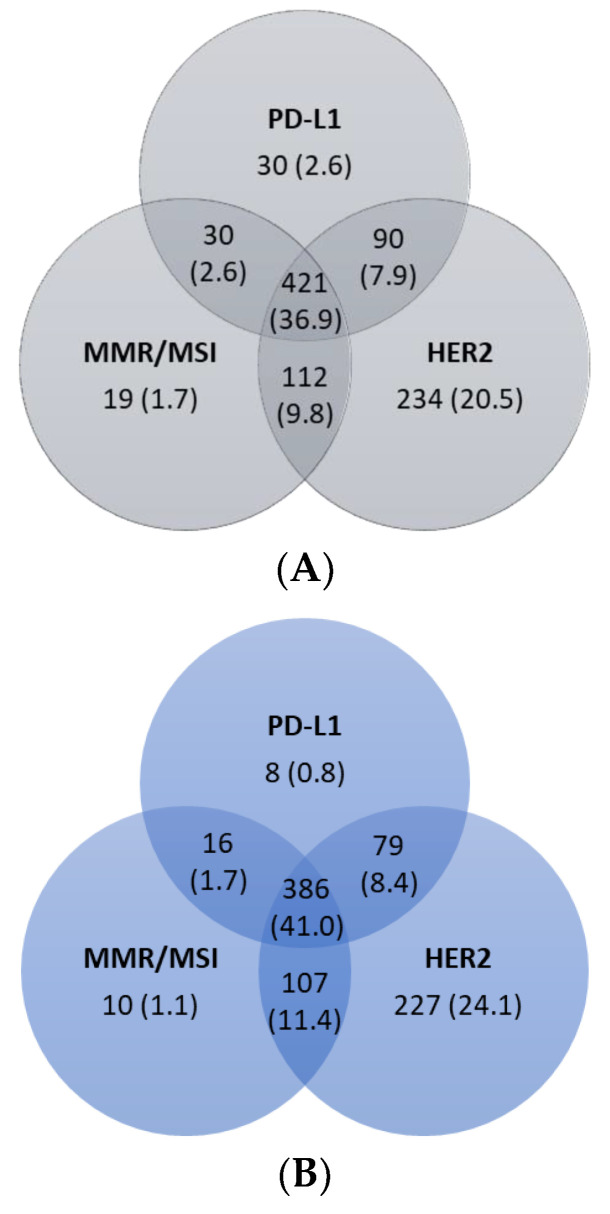
Biomarker testing combinations. Venn diagram depicting biomarker testing combinations for PD-L1, MMR/MSI, and HER2. A: Number (%) of patients tested for PD-L1, MMR/MSI, and HER2. B: Number (%) of patients tested for PD-L1, MMR/MSI, and HER2. HER2, human epidermal growth factor receptor 2. MSI, microsatellite instability. MMR, mismatch repair. PD-L1, programmed cell death-ligand 1. (**A**) Overall (N = 1142); (**B**) patients with adenocarcinomas (N = 942).

A total of 421 (37%) patients were tested for PD-L1, MMR/MSI, and HER2 (Figure 3A). Of the 942 patients with adenocarcinomas, 386 (41%) patients were tested for all three biomarkers (Figure 3B).

Of the 451 patients tested for PD-L1 and MMR/MSI, 5 (1%) patients were classified as both PD-L1-positive and dMMR/MSI-H. Of the 511 patients tested for PD-L1 and HER2, 18 (4%) tested positive for both. Only one patient (<1%) in the cohort was classified as HER2-positive and dMMR/MSI-H.

### 3.3. Biomarker Testing Rates by Primary Tumor Site and Histology

Overall, fewer patients with squamous histology underwent biomarker testing.

Of the 361 patients with esophageal adenocarcinoma, 187 (52%) were tested for PD-L1, 194 (54%) were tested for MMR/MSI, and 302 (84%) were tested for HER2. A total of 42 (22%) patients were PD-L1-positive, 9 (5%) were dMMR/MSI-H, and 94 (31%) were HER2-positive.

Of the 172 patients with esophageal squamous cell carcinoma, 67 (39%) were tested for PD-L1, 49 (28%) were tested for MMR/MSI, and 43 (25%) were tested for HER2 (Table 1 and Table S2). A total of 13 (19%) patients were PD-L1-positive, none were dMMR/MSI-H, and 3 (7%) were HER2-positive.

Of the 337 patients with gastric adenocarcinoma, 180 (53%) were tested for PD-L1, 199 (59%) were tested for MMR/MSI, and 295 (88%) were tested for HER2 (Table 1 and Table S2). A total of 41 (23%) patients were PD-L1-positive, 16 (8%) were dMMR/MSI-H, and 42 (14%) were HER2-positive.

Of the 244 patients with GEJ adenocarcinoma, 122 (50%) were tested for PD-L1, 126 (52%) were tested for MMR/MSI, and 202 (83%) were tested for HER2 (Table 1 and Table S2). Twenty (16%) patients were PD-L1-positive, 5 (4%) were dMMR/MSI-H, and 59 (29%) were HER2-positive.

### 3.4. Treatment Sequences

**Figure 4:**
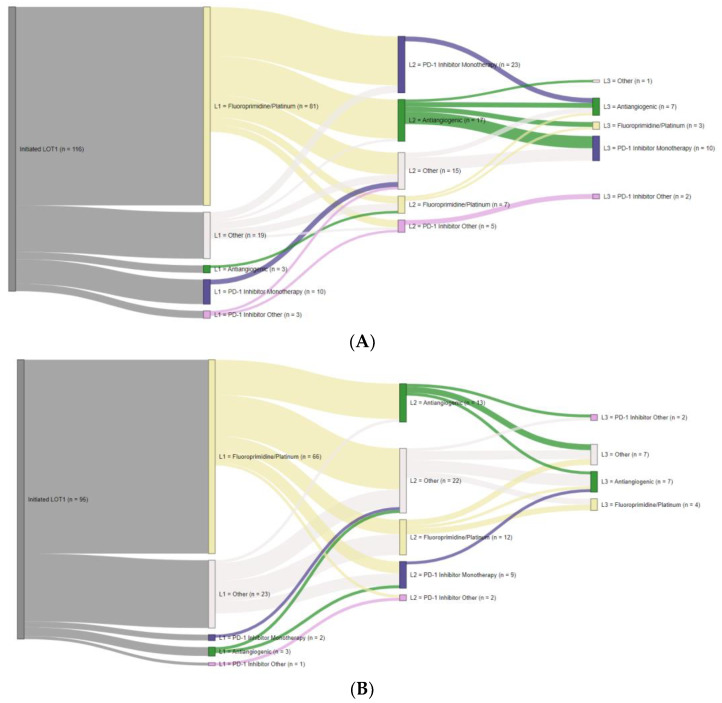

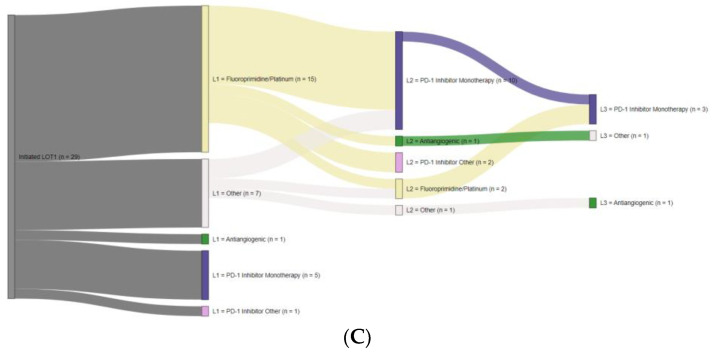
Sankey diagrams for treatment sequences. Patients whose only regimen was weekly carboplatin and paclitaxel are not included in Sankey diagrams. L1, first-line. L2, second-line. L3, third-line. LOT, line of therapy. MSI, microsatellite instability. MMR, mismatch repair. n, number of patients tested. PD-1, programmed death receptor-1. PD-L1, programmed cell death-ligand 1. (**A**) PD-L1-positive; (**B**) PD-L1-negative; (**C**) dMMR/MSI-H.

Nearly 50% of patients with positive PD-L1 results received a PD-1 inhibitor. These patients more commonly received PD-1 monotherapy in second-line (2L) than third-line (3L; Figure 4A). Median (IQR) time on a 2L PD-1 inhibitor was 2.3 (0.8–3.7) months. Fifteen patients with negative PD-L1 results received a PD-1 inhibitor. These patients more commonly received PD-1 monotherapy in 2L, with a median (IQR) time on a 2L PD-1 inhibitor of 3.8 (3.1–7.6) months (Figure 4B). Approximately one-third of patients with unknown results received a PD-1 inhibitor, also more commonly as monotherapy and in 2L (Figure S3B). Median (IQR) time on a 2L PD-1 inhibitor was 2.1 (<0.1–4.4) months. PD-1 inhibitors were rarely used among patients with no PD-L1 testing (Figure S3B).

Of the 30 patients with dMMR/MSI-H results, 20 (>66%) received a PD-1 inhibitor. Six patients received a 1L PD-1 inhibitor, more commonly as monotherapy. No subsequent LOTs were observed, and the median time on a PD-1 inhibitor was not able to be estimated. Twelve patients received a 2L PD-1 inhibitor, also more commonly as monotherapy (Figure 4C). Median (IQR) time on a 2L PD-1 inhibitor was 5.1 (1.9–9.5) months.

Approximately two-thirds of patients with positive HER2 results received a 1L regimen containing a HER2-targeted agent, fluoropyrimidine, and platinum (Figure S3C). Median (IQR) time on 1L HER2-targeted therapy was 7.9 (3.5–14.5) months. Of the patients who received a 2L regimen, >50% received a regimen containing a HER2-targeted agent.

Of the 18 patients with both positive HER2 and PD-L1 results, the majority received a 1L regimen containing a HER2-targeted agent, fluoropyrimidine, and platinum and approximately 50% received 2L or 3L pembrolizumab. No patients received a regimen containing both a HER2-targeted agent and a PD-1 inhibitor. 

## 4. Discussion

This study described real-world testing patterns and results for PD-L1, MMR/MSI, and HER2 in patients with advanced GECs in the United States, primarily in the community setting. The start of the study period coincides with initial recommendations for PD-L1 and MMR/MSI testing while recommendations for HER2 testing had been previously established. Over the study period, PD-L1 and MMR/MSI testing rates increased, and the time between initial diagnosis and the first biomarker test shortened. Despite the potential significance of MSI status as a prognostic predictor in advanced GECs, by 2020, >40% of patients were still not being tested for MMR/MSI. In contrast, nearly 83% of patients with adenocarcinoma were being tested for the well-established HER2 biomarker, a vast majority within a month of initial diagnosis. 

Our study cohort included esophageal, GEJ, and gastric primary tumor sites and was not limited by histology. Adenocarcinoma is the most common type of GEC histology [7,8], while squamous cell carcinoma accounts for <30% of esophageal cancers in the United States [7]. The results of our study are consistent with these reports, with adenocarcinoma as the most prevalent histological type. As expected, biomarker testing rates in adenocarcinomas were higher than those in squamous cell carcinomas. The dMMR/MSI-H results were only observed in adenocarcinomas. Across the study cohort, CPS varied by primary tumor site and histology. Of note, we observed considerable HER2 testing in patients with squamous cell histology, despite the reportedly low incidence of HER2 positivity in esophageal squamous cell carcinoma [24]. At this time, only biomarkers associated with PD-1 inhibitor therapy are recommended for esophageal squamous cell carcinoma [7], but perhaps other targets will be identified to improve outcomes.

PD-L1 expression has been reported in >59% of gastric cancer patients [25], while CPS ≥1 was reported in >80% of patients in the CHECKMATE-649 trial [18]. The PD-L1-positivity rate in patients with gastric/GEJ adenocarcinoma in this study was approximately 20%. While the proportion of tests with unknown results was high, nearly 80% of PD-L1-tested patients with gastric/GEJ adenocarcinoma had CPS ≥1 when reported. Eligibility criteria and additional testing could possibly explain higher PD-L1-positivity rates in clinical trials.

Between 9% and 22% of gastric cancers are diagnosed with dMMR [26]. MSI-H expression ranged from 3 to 7% in the CHECKMATE-649, KEYNOTE-062, and KEYNOTE-059 trials [18,27,28]. The dMMR/MSI-H rates among gastric cancer patients in this study are in line with previous findings. 

HER2-positivity rates range from 12 to 23% in patients with gastric cancer [8]. HER2-positive tumors were seen in 16–24% of patients in the KEYNOTE-811 and DESTINY-Gastric01 trials [18,29,30]. In the present study, HER2-positivity rates among patients with gastric cancer were consistent with these reports.

Some interesting findings related to the use of PD-1 inhibitors in GECs could be seen in this study. Of note, during the study period, 3L pembrolizumab was approved for use in gastric/GEJ adenocarcinoma with CPS ≥ 1 [15]. In this study, PD-1 inhibitor use was more common in 2L and among patients with positive PD-L1 results relative to those with negative or unknown results. Two-thirds of patients with dMMR/MSI-H results received a PD-1 inhibitor, most commonly as monotherapy. With the growing body of evidence of efficacy with PD-1 inhibitors in this subset of patients, we hope that in the future, all patients with dMMR/MSI-H status will receive PD-1 inhibitors unless contraindicated. Regardless of the testing pattern, the uptake of HER2-targeted therapy in 1L was not 100%, especially considering that the treatment paradigm had been available for over 5 years at the time of the start of this study. Moreover, the use of HER2-targeted therapy in 2L is also unconventional since its use beyond progression in GECs has been controversial due to resistance patterns [31]. 

Lack of biomarker testing documentation or out-of-network treatment is a potential limitation of the study. LOT derivations may not accurately represent treatment regimens. The study period (January 2017 to August 2020) preceded a change in treatment paradigms associated with the approval of PD-1 inhibitors in 1L regimens. Similarly, initial recommendations for PD-1 inhibitor use for dMMR/MSI-H were for later lines, but data are now increasing for use in earlier lines [14].

Strengths of the study are the large sample population, the availability of a biomarker database, and the multiple institutions. Data abstraction was conducted by trained personnel following strict procedures. Cohort characteristics are representative of patients with advanced GECs.

## 5. Conclusions

This study describes potential gaps in existing biomarker testing in routine clinical practice for patients with advanced GECs. Biomarker testing results in real-world practice are generally consistent with clinical trial findings. While real-world GEC biomarker testing patterns are not perfect, testing is being conducted soon after diagnosis. Moreover, with newer biomarkers such as Claudin 18.2 and FGFR2b coming into this space [32], the approach to biomarker testing in the future is likely to shift. With recent changes in approvals for PD-1 inhibitors, it will be interesting to see how testing practices and treatment patterns evolve, and increased biomarker testing is expected to enhance treatment decision-making.

## Figures and Tables

**Table 1:** Baseline clinical and disease-related characteristics by PD-L1 and MMR/MSI testing status.

	PD-L1 (N = 1142)		MMR/MSI (N = 1142)	
Variable	PD-L1 Tested (N = 571)	PD-L1 Not Tested (N = 571)	MMR/MSI Tested (N = 582)	MMR/MSI Not Tested (N = 560)
**Diagnosed initially with advanced disease (*n* (%))**	489 (85.6)	511 (89.5)	498 (85.6)	502 (89.6)
**ECOG performance status at 1L start (*n* (%))**				
0	178 (31.2)	161 (28.2)	184 (31.6)	155 (27.7)
1	202 (35.4)	184 (32.2)	211 (36.3)	175 (31.3)
2	55 (9.6)	60 (10.5)	52 (8.9)	63 (11.3)
3	16 (2.8)	20 (3.5)	15 (2.6)	21 (3.8)
4	0	2 (0.4)	0	2 (0.4)
Not reported	80 (14.0)	104 (18.2)	75 (12.9)	109 (19.5)
**Primary tumor site and histology (*n* (%)) ^a^**				
**Esophageal**	*n* = 258	*n* = 283	*n* = 248	*n* = 293
Adenocarcinoma	187 (72.4)	174 (61.4)	194 (78.2)	167 (56.9)
Adenosquamous	2 (0.7)	2 (0.7)	2 (0.8)	2 (0.6)
Squamous cell carcinoma	67 (25.9)	105 (37.1)	49 (19.7)	123 (41.9)
Other	2 (0.7)	2 (0.7)	3 (1.2)	1 (0.3)
**Gastric**	*n* = 187	*n* = 159	*n* = 203	*n* = 143
Adenocarcinoma	180 (96.2)	157 (98.7)	199 (98.0)	138 (96.5)
Adenosquamous	1 (0.5)	0	1 (0.4)	0
Other	6 (3.2)	2 (1.2)	3 (1.4)	5 (3.4)
**Gastroesophageal junction**	*n* = 126	*n* = 129	*n* = 131	*n* = 124
Adenocarcinoma	122 (96.8)	122 (94.5)	126 (96.1)	118 (95.1)
Adenosquamous	0	1 (0.7)	1 (0.7)	0
Squamous cell carcinoma	1 (0.7)	6 (4.6)	1 (0.7)	6 (4.8)
Other	3 (0.2)	0	3 (2.2)	0
**Follow-up from 1L start, months (median (IQR))**	10 (6, 15)	9 (6, 15)	10 (6, 15)	9 (6, 14)

^a^ Percentage based on primary tumor site. 1L, first-line. ECOG, Eastern Cooperative Oncology Group. IQR, interquartile range. MSI, microsatellite instability. MMR, mismatch repair. N, total number of patients. *n*, number of patients. PD-L1, programmed cell death-ligand 1.

## Data Availability

The data that support the findings of this study have been originated by Flatiron Health, Inc. These de-identified data may be made available upon request and are subject to a license agreement with Flatiron Health; interested researchers should contact DataAccess@flatiron.com to determine licensing terms.

## Supplementary Materials

The following supporting information can be downloaded at: https://www.mdpi.com/article/10.3390/curroncol30020145/s1, Figure S1: Attrition flowchart; Figure S2: Biomarker testing rates and results; Figure S3: Sankey diagram for treatment sequences; Table S1: Baseline demographic characteristics by PD-L1 and MMR/MSI testing status; Table S2: Baseline demographic characteristics by HER2 testing status; Table S3: Baseline demographics and testing characteristics by NGS testing status; Table S4: Overall biomarker test counts, tissue collection sites, and assays; Table S5: Details on first two biomarker tests in patients who underwent multiple biomarker tests; Table S6: PD-L1 combined positive scores overall and by primary tumor site; Table S7: Baseline clinical and disease-related characteristics by HER2 testing status.
