# Supplementary material for: Real-World Molecular Biomarker Testing Patterns and Results for Advanced Gastroesophageal Cancers in the United States
Source: Curr Oncol. 2023 Feb 3;30(2):1869–81. doi: 10.3390/curroncol30020145 (PMC9955769; doi:10.3390/curroncol30020145)
Supplement: Supplementary file 1 [file curroncol-30-00145-s001.zip › curroncol-2141168-supplementary.pdf]

Supplementary data

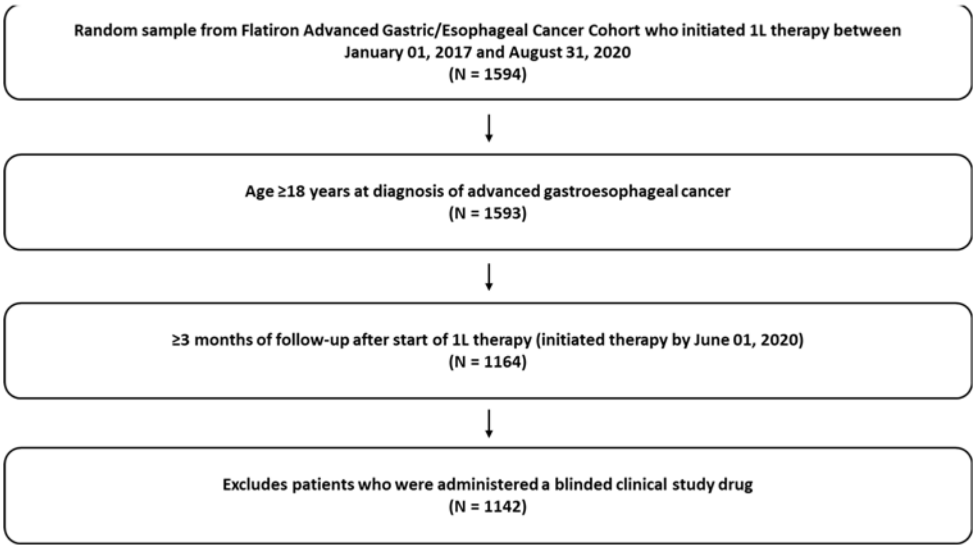

Figure S1. Attrition Flowchart. 1L, first-line. N, total number of patients.

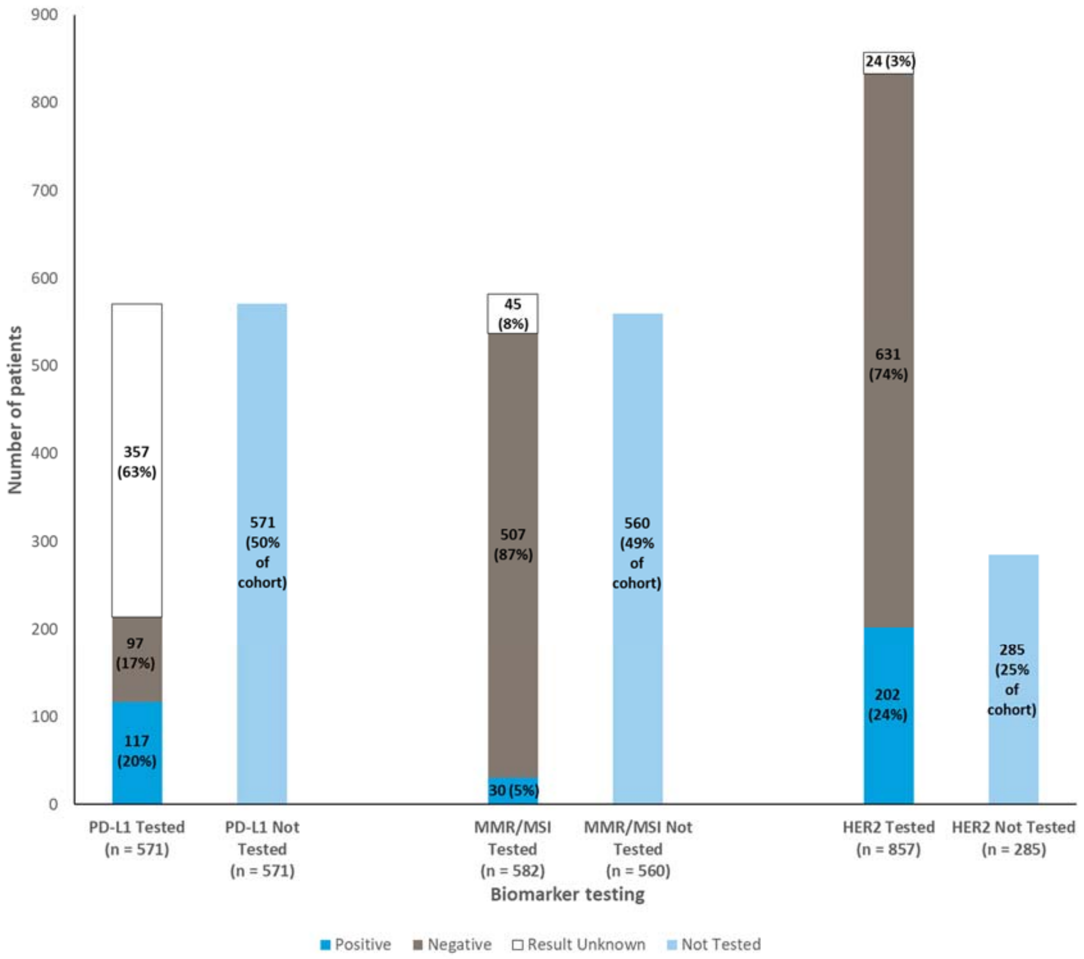

Figure S2. Biomarker Testing Rates and Results. dMMR, deficient mismatch repair. HER2, human epidermal growth factor receptor 2. MSI, microsatellite instability. MSI-H, microsatellite instability-high. MMR, mismatch repair. n, number of patients. PD-L1, programmed cell death-ligand 1.

A. PD-L1-unknown

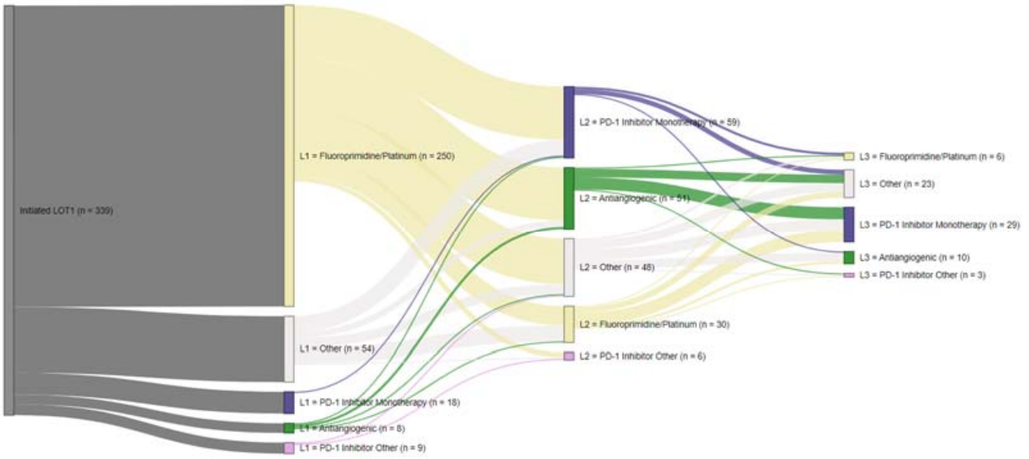

B. PD-L1 Not Tested

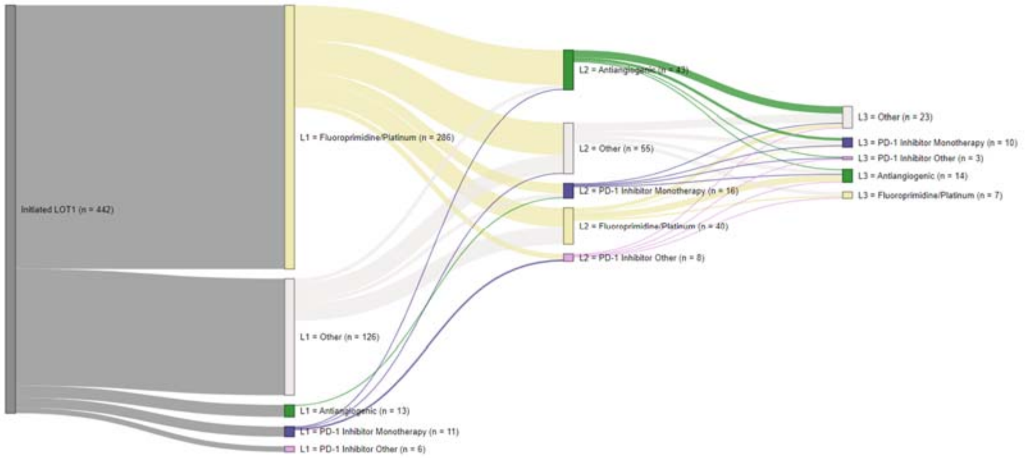

C. HER2-positive

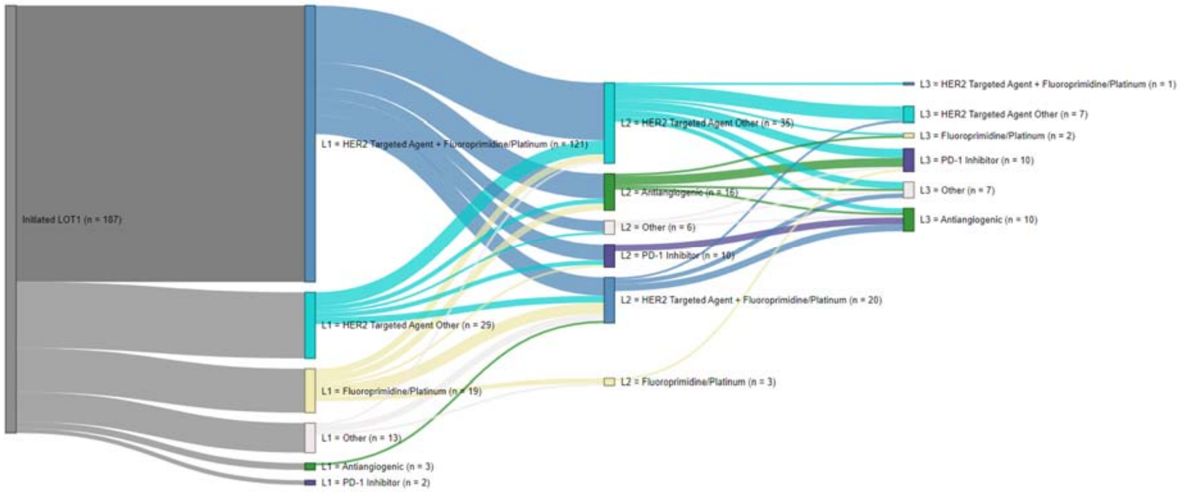

Figure S3: Sankey diagram for treatment sequences. Patients whose only regimen was weekly carboplatin and paclitaxel are not included in Sankey diagrams. A: PD-L1 unknown. B: PD-L1 not tested. C: HER2-positive. L1, first-line. L2, second-line. L3, third-line. HER2, human epidermal growth factor receptor 2. LOT, line of therapy. PD-1, programmed death receptor-1. PD-L1, programmed cell death-ligand 1.

Table S1. Baseline demographic characteristics by PD-L1 and MMR/MSI testing status

	**Overall (N=1142)**	**PD-L1 (N=1142)**		**MMR/MSI (N=1142)**	
**Variable**		**Tested (N=571)**	**Not tested (N=571)**	**Tested (N=582)**	**Not tested (N=560)**
**Age in years at start of 1L therapy, (median [range])**	Range 23–84	67 (27–84)	70 (23–84)	66 (27–84)	70 (23–84)
**Sex, (n [%])**					
Female	286 (25.0)	149 (26.1)	137 (24.0)	149 (25.6)	137 (24.5)
Male	856 (74.9)	422 (73.9)	434 (76.0)	433 (74.4)	423 (75.5)
**Race, (n [%])**					
White	725 (63.5)	348 (61.0)	377 (66.0)	362 (62.2)	363 (64.8)
Black/African American	87 (7.62)	39 (6.8)	48 (8.4)	39 (6.7)	48 (8.6)
Asian	33 (2.9)	20 (3.5)	13 (2.3)	19 (3.3)	14 (2.5)
Other	157 (13.7)	84 (14.7)	73 (12.8)	83 (14.3)	74 (13.2)
Missing	140 (12.3)	80 (14.0)	60 (10.5)	79 (13.6)	61 (10.9)
**Hispanic/Latino, (n [%])**	92 (8.0)	38 (6.7)	54 (9.5)	43 (7.4)	49 (8.8)
**Geographic region, (n [%])**					
Midwest	159 (13.9)	81 (14.2)	78 (13.7)	86 (14.8)	73 (13.0)
Northeast	212 (18.6)	123 (21.5)	89 (15.6)	114 (19.6)	98 (17.5)
South	454 (39.8)	218 (38.2)	236 (41.3)	216 (37.1)	238 (42.5)
West	214 (18.7)	112 (19.6)	102 (17.9)	124 (21.3)	90 (16.1)
Unknown	103 (9.0)	37 (6.5)	66 (11.6)	42 (7.2)	61 (10.9)
**Practice type, (n [%])**					
Academic	93 (8.1)	29 (5.1)	64 (11.2)	36 (6.2)	57 (10.2)
Community	1049 (91.9)	542 (94.9)	507 (88.8)	546 (93.8)	503 (89.8)
**Payer category, (n [%])**					
Commercial health plan	488 (42.7)	260 (45.5)	228 (39.9)	254 (43.6)	234 (41.8)
Medicare	168 (14.7)	68 (11.9)	100 (17.5)	69 (11.9)	99 (17.7)
Medicaid or other government program	74 (6.5)	40 (7.0)	34 (6.0)	40 (6.9)	34 (6.1)
Patient assistance program	75 (6.6)	47 (8.2)	28 (4.9)	60 (10.3)	15 (2.7)
Other payer – type unknown	304 (26.6)	141 (24.7)	163 (28.6)	142 (24.4)	162 (28.9)
Self-pay	14 (1.2)	6 (1.1)	8 (1.4)	7 (1.2)	7 (1.3)

1L, first-line. MSI, microsatellite instability. MMR, mismatch repair. N, total number of patients. n, number of patients. PD-L1, programmed cell death-ligand 1.

Table S2. Baseline demographic characteristics by HER2 testing status

	HER2 (N=1142)	
**Variable**	**Tested (N=857)**	**Not tested (N=285)**
**Age in years at start of 1L therapy, (median [range])**	67 (23–84)	70 (38–84)
**Sex, (n [%])**		
Female	208 (24.2)	78 (27.3)
Male	649 (75.7)	207 (72.6)
**Race, (n [%])**		
White	539 (62.8)	186 (65.2)
Black/African American	58 (6.7)	29 (10.1)
Asian	24 (2.8)	9 (3.1)
Other	126 (14.7)	28 (9.8)
Missing	107 (12.4)	60 (10.5)
**Hispanic/Latino, (n [%])**	72 (8.4)	20 (7.0)
**Geographic region, (n [%])**		
Midwest	128 (14.9)	31 (10.8)
Northeast	160 (18.6)	52 (18.2)
South	330 (38.5)	124 (43.5)
West	165 (19.2)	49 (17.1)
Unknown	74 (8.6)	29 (10.1)
**Practice type, (n [%])**		
Academic	66 (7.7)	27 (9.4)
Community	791 (92.3)	258 (90.5)
**Payer category, (n [%])**		
Commercial health plan	367 (42.8)	121 (42.5)
Medicare	124 (14.4)	44 (15.4)
Medicaid or other government program	55 (6.4)	19 (6.7)
Patient assistance program	59 (6.8)	16 (5.6)
Self-pay or other payer–type unknown	237 (27.7)	81 (28.4)

1L, first-line. HER2, human epidermal growth factor receptor 2. N, total number of patients. n, number of patients.

Table S3. Baseline demographics and testing characteristics by NGS testing status

	NGS (N=1142)	
**Variable**	**Tested (N=394)**	**Not tested (N=748)**
**Age in years at start of 1L therapy, (median [IQR])**	65 (57, 72)	70 (62, 77)
**Sex, (n [%])**		
Female	100 (25.3)	186 (24.8)
Male	294 (74.6)	562 (75.1)
**Race, (n [%])**		
White	247 (62.6)	478 (63.9)
Black/African American	25 (6.3)	62 (8.2)
Asian	12 (3.0)	21 (2.8)
Other	63 (15.9)	91 (12.1)
Missing	45 (11.4)	95 (12.7)
**Hispanic/Latino**	31 (7.8)	61 (8.1)
**Geographic region, (n [%])**		
Midwest	69 (17.5)	90 (12.0)
Northeast	70 (17.7)	142 (18.9)
South	141 (35.7)	313 (41.8)
West	80 (20.3)	134 (17.9)
Unknown	34 (8.6)	69 (9.2)
**Practice type, (n [%])**		
Academic	30 (7.6)	63 (8.4)
Community	364 (92.3)	685 (91.5)
**Payer category, (n [%])**		
Commercial health plan	174 (44.1)	314 (41.9)
Medicare	51 (12.9)	117 (15.6)
Medicaid or other government program	23 (5.8)	51 (6.8)
Patient assistance program	43 (10.9)	32 (4.2)
Self-pay or other payer – type unknown	97 (24.6)	221 (29.5)
**Number of tests per patient, (n [%])**		
0	0	748 (100)
1	341 (86.5)	—
2	46 (11.7)	—
3	4 (1.0)	—
4	2 (<1)	—
5	1 (<1)	—

1L, first-line. IQR, interquartile range. N, total number of patients. n, number of patients. NGS, next-generation sequencing.

Table S4. Overall biomarker test counts, tissue collection sites, and assays

	PD-L1		MMR/MSI		HER2	
**Variable**	**Tested (N=571)**	**Not tested (N=571)**	**Tested (N=582)**	**Not tested (N=560)**	**Tested (N=857)**	**Not tested (N=285)**
**Number of tests per patient, (n[%])**						
0	0	571 (100)	0	560 (100)	0	285 (100)
1	480 (84.1)	—	386 (66.3)	—	461 (53.8)	—
2	64 (11.2)	—	155 (26.6)	—	278 (32.4)	—
3	23 (4.0)	—	30 (5.2)	—	73 (8.5)	—
4	4 (<1)	—	8 (1.4)	—	32 (3.7)	—
5	0	—	1 (<1)	—	10 (1.2)	—
6	0	—	2 (<1)	—	0	—
7	0	—	0	—	2 (<1)	—
8	0	—	0	—	1 (<1)	—
**Tissue collection site, (n[%])***	N=693		N=835		—	
Metastatic	169 (24.4)		217 (26.0)		—	
Primary	517 (74.6)		568 (68.0)		—	
Unknown	7 (1.0)		50 (6.0)		—	
**Assays used to perform tests, (n[%])**	N=693		N=835		N=1436	
Dako PD-L1 IHC 22C3 pharmDX	490 (70.7)		—		—	
Laboratory-developed tested	74 (10.7)		—		—	
Ventana PD-L1 (SP142)	20 (2.9)		—		—	
Ventana PD-L1 (SP263)	13 (1.9)		—		—	
IHC	—		384 (46.0)		798 (55.6)	
NGS	—		358 (42.9)		158 (11.0)	
PCR	—		65 (7.8)		—	
FISH	—		—		393 (27.4)	
Unknown	96 (13.9)		28 (3.4)		87 (6.1)	

*Information on tissue collection site for HER2 was not available in the database. FISH, fluorescence in situ hybridization. HER2, human epidermal growth factor receptor 2. IHC, immunohistochemistry. MSI, microsatellite instability. MMR, mismatch repair. N, total number of patients. n, number of patients. NGS, next-generation sequencing. PCR, polymerase chain reaction. PD-L1, programmed cell death-ligand 1.

Table S5. Details on first two biomarker tests in patients who underwent multiple biomarker tests

	PD-L1 Tested		MMR/MSI Tested		HER2 Tested	
**Variable**	**Patients With Same Results (n=59)**	**Patients With Different Results (n=32)**	**Patients With Same Results (n=158)**	**Patients With Different Results (n=38)**	**Patients With Same Results (n=241)**	**Patients With Different Results (n=155)**
**Same assay type, (n [%])**						
Yes	53 (89.8)	26 (81.3)	35 (22.2)	14 (36.8)	57 (23.7)	6 (3.9)
No	6 (10.2)	6 (18.8)	123 (77.9)	24 (63.2)	184 (76.4)	149 (96.1)
**Same tissue collection site (primary or metastatic), (n [%])***						
Yes	49 (83.1)	20 (63.0)	137 (86.7)	26 (68.4)	—	—
No	10 (17.0)	12 (38.0)	21 (13.3)	12 (31.6)	—	—
**Days between tests, (n [%])**						
Same day	43 (72.9)	19 (59.4)	117 (74.1)	20 (52.6)	156 (64.7)	124 (80.0)
1-29 days	6 (10.2)	1 (3.1)	13 (8.2)	10 (26.3)	22 (9.1)	9 (5.8)
30-90 days	—	—	3 (2.0)	1 (2.6)	7 (3.0)	4 (2.6)
>90 days	10 (17.0)	12 (37.5)	25 (15.8)	7 (18.4)	56 (23.2)	18 (11.6)
**Initiated a new LOT with PD-1 inhibitor between tests, (n [%])**	0	0	1 (4.4)	2 (18.2)	—	—
**Initiated a new LOT with HER2-targeted agent between tests, (n [%])**	—	—	—	—	4 (12.1)	5 (45.5)

*Information on tissue collection site for HER2 was not available in the database. HER2, human epidermal growth factor receptor 2. LOT, line of therapy. MSI, microsatellite instability. MMR, mismatch repair. N, total number of patients. n, number of patients. PD-1, programmed cell death receptor-1. PD-L1, programmed cell death-ligand 1.

Table S6. PD-L1 combined positive scores overall and by primary tumor site

	0	<1	1	2-4	5-9	10-19	20-29	≥30	Total CPS reported	CPS Not Reported
**Overall, (n [%])**	48 (11.8)	30 (7.4)	67 (16.5)	61 (15.0)	65 (16.0)	64 (15.8)	30 (7.4)	41 (10.1)	406	165
**Esophageal, (n [%])**										
Adenocarcinoma	16 (11.5)	10 (7.2)	24 (17.3)	29 (20.9)	15 (10.8)	21 (15,1)	12 (8.6)	12 (8.6)	139	48
Adenosquamous	—	—	1 (50)	—	—	—	—	1 (50)	2	—
Squamous cell carcinoma	3 (8.6)	—	2 (5.7)	1 (2.6)	1 (2.6)	11 (31.4)	5 (14.3)	12 (34.3)	35	32
Other	—	—	—	1 (50)	—	1 (50)	—	—	2	—
**Gastric, (n [%])**										
Adenocarcinoma	12 (9.4)	7 (5.5)	27 (21.1)	20 (15.6)	34 (26.6)	14 (10.9)	5 (3.9)	9 (7.0)	128	52
Adenosquamous	—	—	—	—	—	—	—	—	—	1
Other	1 (33.3)	—	—	—	—	1 (33.3)	—	1 (33.3)	3	3
**Gastroesophageal junction, (n [%])**										
Adenocarcinoma	16 (16.7)	13 (13.5)	13 (13.5)	10 (10.4)	15 (15.6)	16 (16.7)	8 (8.3)	5 (5.2)	96	26
Squamous cell carcinoma	—	—	—	—	—	—	—	1 (100)	1	—
Other	—	—	—	—	—	—	—	—	—	3

Note: Percentages are based on total CPS reported results. CPS, combined positive scores. n, number of patients. PD-L1, programmed cell death-ligand 1.

Table S7. Baseline clinical and disease-related characteristics by HER2 testing status

	HER2 (N=1142)	
**Variable**	**Tested (N=857)**	**Not tested (N=285)**
**Patients diagnosed initially with advanced disease, n (%)**	734 (85.6)	266 (93.3)
**ECOG performance status, (n [%])**		
0	255 (29.7)	84 (29.4)
1	294 (34.3)	92 (32.2)
2	82 (9.5)	33 (11.5)
3	28 (3.2)	8 (2.8)
4	2 (0.2)	0
Not reported	129 (15.0)	55 (19.3)
**Primary tumor site and Histology, (n [%])[a]**		
**Esophageal**	n=350	n=191
Adenocarcinoma	302 (86.3)	59 (30.9)
Adenosquamous	3 (0.8)	1 (0.5)
Squamous cell carcinoma	43 (12.3)	129 (67.5)
Other	2 (0.6)	2 (1.0)
**Gastric**	n=301	n=45
Adenocarcinoma	295 (98.0)	42 (93.3)
Adenosquamous	1 (0.3)	0
Other	5 (1.7)	3 (0.7)
**Gastroesophageal junction**	n=206	n=49
Adenocarcinoma	202 (98.0)	42 (85.7)
Adenosquamous	1 (0.5)	0
Squamous cell carcinoma	—	7 (14.3)
Other	3 (1.5)	—
**Follow-up from 1L start, months, (median [IQR])**	9 (6, 15)	10 (6, 15)

[a]Percentages based on primary tumor site. 1L, fist-line. HER2, human epidermal growth factor receptor 2. ECOG, Eastern Cooperative Oncology Group. IQR, interquartile range. N, total number of patients. n, number of patients.
